# Association of combustible cigarettes and heated tobacco products use with SARS-CoV-2 infection and severe COVID-19 in Japan: a JASTIS 2022 cross-sectional study

**DOI:** 10.1038/s41598-023-28006-3

**Published:** 2023-02-02

**Authors:** Misako Nishimura, Kazuhisa Asai, Takahiro Tabuchi, Erika Toyokura, Takahiro Kawai, Atsushi Miyamoto, Tetsuya Watanabe, Tomoya Kawaguchi

**Affiliations:** 1 Department of Respiratory Medicine, Graduate School of Medicine, Osaka City University, 1-4-3, Asahimachi, Abeno-Ku, Osaka 545-8585 Japan; 2 Department of Respiratory Medicine, Graduate School of Medicine, Osaka Metropolitan University, 1-4-3, Asahimachi, Abeno-Ku, Osaka 545-8585 Japan; 3 Cancer Control Center, Osaka International Cancer Institute, 1-69, Ohtemae 3-Chome, Chuo-Ku, Osaka 541-8567 Japan

**Keywords:** Epidemiology, Risk factors, Viral infection

## Abstract

Insufficient evidence has been accumulated regarding associations of heated tobacco products (HTPs) use with coronavirus infection and severity of coronavirus disease 2019 (COVID-19), an ongoing pandemic. We conducted a cross-sectional study using data from an internet questionnaire administered in February 2022 to 30,130 individuals from the general Japanese population (age range, 16–81 years). Single users of HTPs and dual users of combustible cigarettes and HTPs comprised 5.2% and 7.3% of respondents, and 6.7% and 38.0% of those infected (n = 1117). Approximately 70% of infected dual users experienced severe disease. Single users of HTPs and dual users were more likely to be infected with coronavirus than never-users (adjusted odds ratio [aOR] = 1.65/4.66; 95% confidence interval [CI] 1.26–2.15/3.89–5.58). Regarding severity, former and current tobacco users (former/combustible cigarettes/HTPs: aOR = 1.88/3.17/1.90; 95%CI 1.11–3.19/1.77–5.67/1.01–3.59) were more likely to be administered oxygen than never-users, and dual users required oxygen administration the most (aOR = 4.15, 95%CI 2.70–6.36). Use of HTPs may increase risks of coronavirus infection and severe COVID-19. Our results provide an opportunity to consider the safety of tobacco products use, including HTPs, during the COVID-19 pandemic.

## Introduction

In recent years, new tobacco products such as electronic cigarettes (e-cigarettes) and heated tobacco products (HTPs) have been developed to reduce exposure to tobacco smoke. E-cigarettes are devices that heat liquid to generate a vapor for inhalation, and are available with or without nicotine^1^. HTPs are devices that generate aerosols by heating tobacco leaves without burning them^2^. In Europe and the Unites States, e-cigarettes have rapidly gained popularity, particularly among young people, including teenagers^1^.

In Japan, nicotine-containing e-cigarettes are not commercially available because nicotine is treated as a prescription drug, while nicotine-free e-cigarettes are not regulated. E-cigarette use in Japan has been gradually increasing^3^; in 2018, about 5% of young adults aged 15–29 years used e-cigarettes^4^. However, the rate of use among the general population is not very high. Meanwhile, HTPs have been approved as tobacco products and marketed in Japan similarly to combustible cigarettes. HTPs are rapidly spreading in Japan and account for about one-third of the tobacco market, as estimated using statistical data from the Tobacco Institute of Japan for 2021^5^.

Combustible cigarettes contain high levels of nicotine, causing dependence, and tar in their smoke, and habitual use can lead to diseases such as pulmonary disease, cardiovascular disease, and malignant tumors of lungs, larynx, esophagus, etc.^6,7^. On the other hand, the effects on health of HTPs themselves remain unclear, because HTPs have only been on the market for a short time and most current users are dual users who also use combustible cigarettes^3,8^. Components in aerosols from HTPs are reported to show slightly lower to similar levels of nicotine and relatively higher levels of propylene glycol, glycerol, and acetol when compared to those of combustible cigarettes^2,9^. Several reports have described users of HTPs developing severe eosinophilic pneumonia after brief use or when changing from combustible cigarettes, as is sometimes seen in those starting combustible cigarettes, and components in aerosols may have contributed to this^10,11^.

Coronavirus disease 2019 (COVID-19) is an ongoing pandemic, sometimes causing severe pneumonia and death, and is a major problem worldwide. COVID-19 is caused by severe acute respiratory syndrome coronavirus 2 (SARS-CoV-2) infection, and angiotensin-converting enzyme 2 expressed in airway epithelium has been reported as the entry receptor^12^. Regarding the risk of SARS-CoV-2 infection, some reports have suggested that use of combustible cigarettes increases susceptibility to the virus by increasing receptor expression^13^, while others have suggested that nicotine downregulates the receptors^14^. Some reports on clinical cases have found that current tobacco use was associated with severe COVID-19^15–17^, while others found no such association^18–20^. Results regarding the association of tobacco use with SARS-CoV-2 infection and COVID-19 severity are thus conflicting.

During the COVID-19 pandemic, tobacco use has reportedly decreased due to the closure of bars and pubs and constraints on the ability to socialize with friends^21^, while use of HTPs has reportedly increased due to switching from combustible cigarettes^22^ and increased demand for tobacco when outings were restricted^23^. However, insufficient evidence has been accumulated regarding the safety of HTPs in the context of COVID-19.

In response to these circumstances, we examined the association of tobacco products use including HTPs with SARS-CoV-2 infection and severe COVID-19. Furthermore, we used data from the general population in the present study because many previous reports have focused on inpatients and have noted low proportions of tobacco users^18^.

## Methods

### Study design and participants

We aimed to examine the associations of tobacco products use including HTPs with both SARS-CoV-2 infection and severe COVID-19. We conducted a cross-sectional study using data from the Japan “Society and New Tobacco” Internet Survey (JASTIS), an annual cohort study on the internet aimed at investigating the effects on health of new tobacco products (HTPs and e-cigarettes) (Study profile is shown in a previous report^24^). Participants in JASTIS were selected from panelists of Rakuten Insight, one of Japan’s largest internet research companies, with 2.2 million users covering various social categories in the general Japanese population (according to education, housing tenure, marital status, etc.). A self-reporting questionnaire was administered on the internet between February 1 and 28, 2022 to randomly sampled panelists by sex and age group at the launch of JASTIS in 2015 and additionally sampled panelists in each year, and the survey was closed when the target number of respondents was reached. Questions consisted of tobacco use status including use of HTPs, history of SARS-CoV-2 infection (within the preceding year/over one year prior to the survey) and severe COVID-19 (hospitalization and oxygen administration), comorbidities, and other background characteristics of the respondents. Respondents answered all items and there were no missing values.

**Figure 1:**
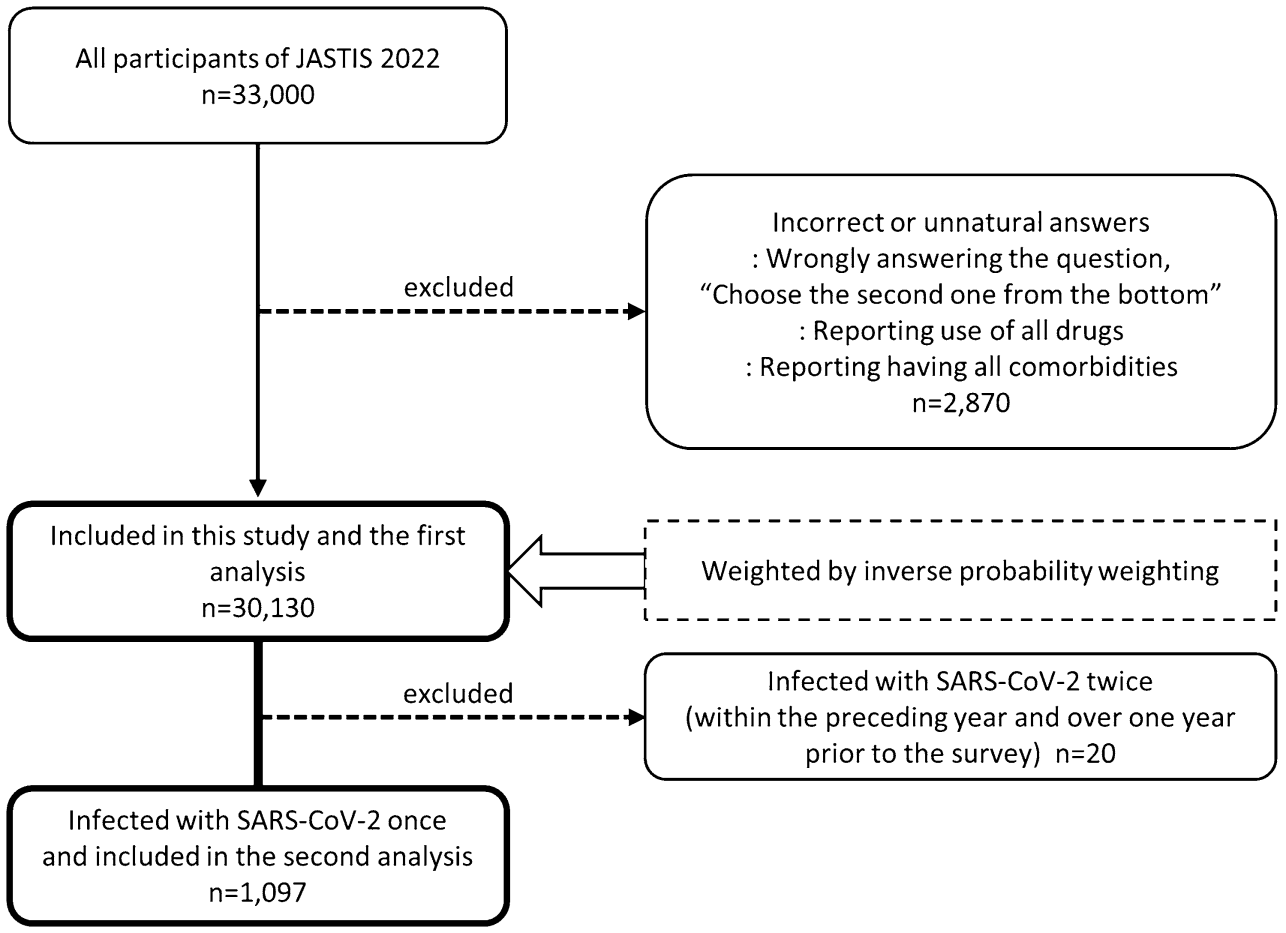
Inclusion flow chart. *JASTIS* the Japan “Society and New Tobacco” Internet Survey, *SARS-CoV-2* severe acute respiratory syndrome coronavirus 2.

Of the 33,000 participants, 30,130 were included in this study and the first analysis (Fig. 1). Those who had been infected with SARS-CoV-2 once were included in the second analysis (n = 1097). The 2870 participants not included in the first analysis were excluded because of incorrect or unnatural answers such as: wrongly answering the question, “Choose the second one from the bottom”; reporting use of all drugs; and reporting having all comorbidities (see details in Supplementary Note). In addition, 20 individuals who reported having been infected twice were excluded from the second analysis to prevent duplicate results.

This study was conducted in accordance with the Ethical Guidelines for Life Science and Medical Research Involving Human Subjects. All study protocols were reviewed and approved by the Research Ethics Committee of the Osaka International Cancer Institute (No. 20084-8). Informed consent was obtained from all participants at the time of study entry by electromagnetic means.

### Definition of variables

**Figure 2:**
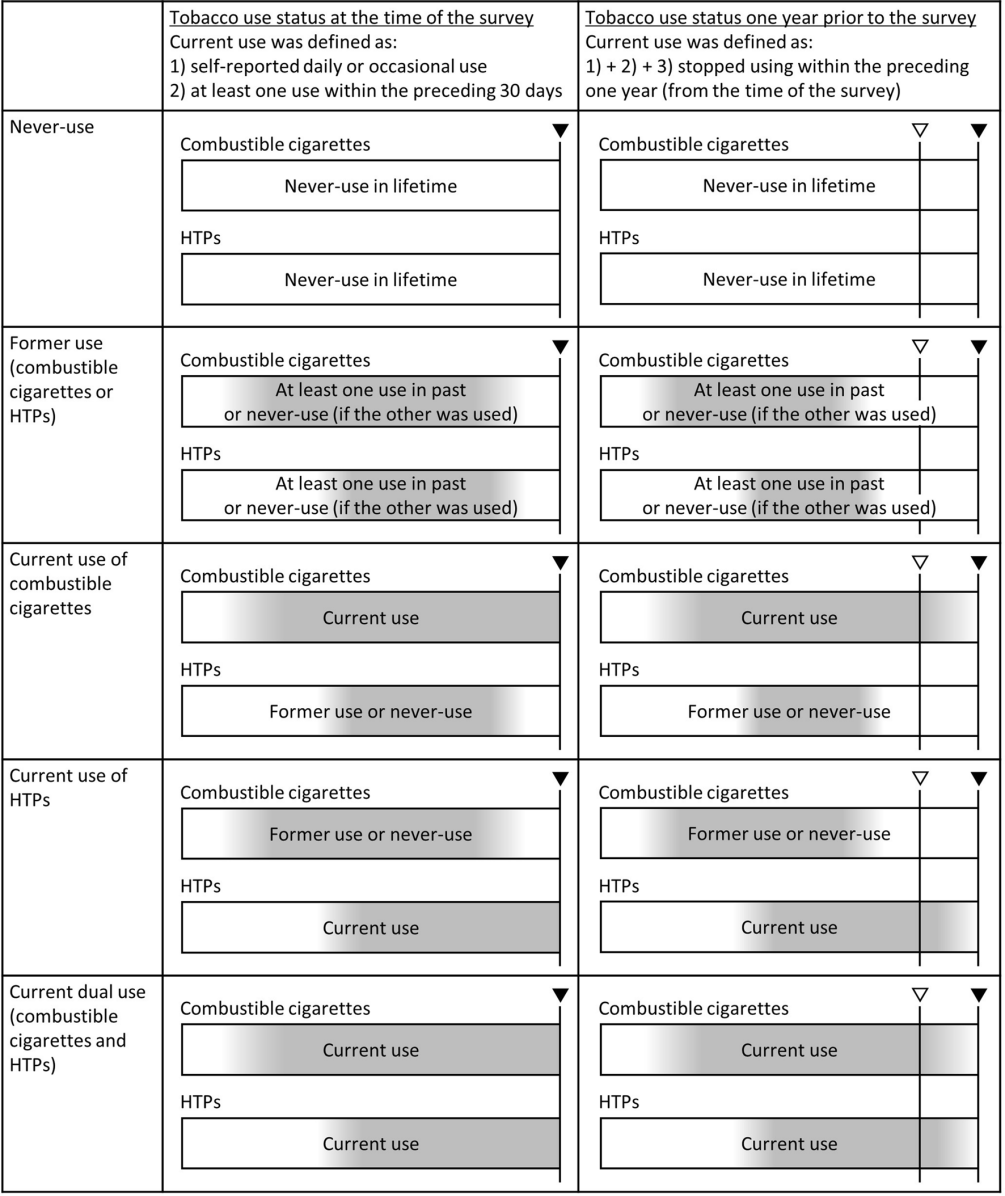
Definition of tobacco use status. Horizontal bars indicate the time course, and gray color indicates the duration of each tobacco use. Inverted triangles indicate the time of the survey (▼) and one year prior to the survey (▽). *HTPs* heated tobacco products.

Tobacco use status was determined based on the respondents’ answers to several questions about use of each tobacco product. Combustible cigarettes were defined as cigarettes and roll-your-own tobacco. HTPs included IQOS, Ploom Tech, Ploom S, Ploom X, glo, and lil HYBRID—brands sold in Japan as of February 2022. The questions and definitions of current tobacco users for each of the above tobacco products were as follows: (1) those who selected 4 or 5 in the question “Do you currently smoke or use tobacco? [(1) Never used before; (2) Tried using more than once, but did not use habitually; (3) Used to use habitually, but stopped; (4) Use occasionally; and (5) Use almost daily]”; (2) those who answered 1 or more to the question “On how many of the preceding 30 days did you smoke or use tobacco? (answer with a whole number between 0 and 30)” (Fig. 2). Particularly for the use of new tobacco products, there is no common historical definition of current use^25^, and Definition 2 was established with reference to previous reports^25,26^. Frequency of use was therefore not considered. Current users in the applicable category (i.e., combustible cigarettes, HTPs, or both) were defined as those who met more than one of the above criteria for any of the tobacco products (e.g., roll-your-own tobacco, IQOS) in the applicable category. Current dual users were defined as those who were currently using both combustible cigarettes and HTPs. Those who reported that they had never used any tobacco were considered as never-users. Those who did not meet any of these definitions were considered as former users. Those who stopped using any tobacco product in the applicable category within the preceding year were included as current users in each category for tobacco use status at one year prior to the survey. Questions about amount of tobacco used per day and year of tobacco cessation did not occur for respondents who were not habitual users (those who answered “2. Tried using more than once, but did not use habitually.” for tobacco use status). Because of the non-negligible missing values for both former users and current users (according to Definition 2), the amount and duration of tobacco use were not included in the analyses in this study.

Regarding SARS-CoV-2 vaccination status, two messenger ribonucleic acid vaccinations or one viral vector type vaccination were defined as two doses, and these plus one booster vaccination were defined as three doses. The history of SARS-CoV-2 infection and severe COVID-19 (hospitalization and oxygen administration) were determined based on the answers to the question of whether the respondent had experienced each, either within the preceding year or over one year prior to the survey.

Details including other items are described in Supplementary Note, Tables S1 and S2.

### Statistical analysis

For the first analysis, we performed multivariable logistic regression analysis to examine the association between tobacco use status and SARS-CoV-2 infection among all participants. We adjusted for the following potential confounding factors: age; sex; occupation; household annual income; education; housing tenure; whether the respondent lived in a prefecture under alert (21 prefectures in which the State of Emergency had been declared more than once, as listed in Supplementary Table S1); presence of household members other than the respondent; whether the respondent frequently interacted with people living separately; total metabolic equivalents (METs) per day; alcohol consumption; comorbidities (12 diseases associated with COVID-19 severity in past reports^16,27^, as listed in Table 1); engagement in infection prevention measures (percentage of 12 infection prevention behaviors preferred in Japan and listed in Supplementary Table S2 engaged in; partially modified with reference to a previous report^28^); and SARS-CoV-2 vaccination status.

**Table 1:** Characteristics of participants.

	All participants (n = 30,130)	Participants infected with SARS-CoV-2 (n = 1117)
Age, n (%)*		
≤ 34 years	8450 (28.0)	657 (58.8)
35–49 years	7696 (25.5)	289 (25.9)
50–64 years	6661 (22.1)	108 (9.7)
≥ 65 years	7323 (24.3)	62 (5.6)
Sex, n (%)		
Male	14,718 (48.8)	714 (63.9)
Female	15,412 (51.2)	403 (36.1)
Tobacco use status, n (%)		
Never-user	16,210 (53.8)	380 (34.0)
Former user (combustible cigarettes or HTPs)	6584 (21.9)	145 (13.0)
Current user of combustible cigarettes	3571 (11.9)	92 (8.2)
Current user of HTPs	1558 (5.2)	75 (6.7)
Current dual user (combustible cigarettes and HTPs)	2206 (7.3)	425 (38.0)
Occupation, n (%)		
Student/unemployed/retiree	10,283 (34.1)	157 (14.1)
Healthcare worker	2265 (7.5)	94 (8.4)
Remote worker	1521 (5.0)	85 (7.6)
Other worker	16,061 (53.3)	781 (69.9)
Household annual income, n (%)		
Below average	14,651 (48.6)	545 (48.8)
Above average (higher)	8392 (27.9)	374 (33.5)
Did not know/did not want to answer	7087 (23.5)	198 (17.7)
Education, n (%)		
Junior high school/high school	15,614 (51.8)	555 (49.7)
University/technical school or more	14,516 (48.2)	561 (50.2)
Housing tenure, n (%)		
Own house	22,665 (75.2)	728 (65.2)
Rental house	6903 (22.9)	359 (32.1)
Other (freeloader/rootless)	562 (1.9)	30 (2.7)
Living in prefectures under alert, n (%)^†^		
No	11,997 (39.8)	315 (28.2)
Yes	18,133 (60.2)	802 (71.8)
Household members other than the participant, n (%)		
None (living alone)	5091 (16.9)	249 (22.3)
Adult(s) only	16,641 (55.2)	425 (38.0)
Living with a child (children)^‡^	8398 (27.9)	443 (39.7)
Interacting with people living separately at least once a week, n (%)		
No	19,453 (64.6)	491 (44.0)
Yes	10,677 (35.4)	625 (56.0)
Total METs per day, n (%)		
Below average	22,489 (74.6)	607 (54.3)
Above average (more active)	7641 (25.4)	510 (45.7)
Alcohol consumption, n (%)		
Never drinker	13,096 (43.5)	394 (35.3)
Ever drinker	17,034 (56.5)	723 (64.7)
Comorbidities, n (%)		
None	18,746 (62.2)	671 (60.1)
One comorbidity	6558 (21.8)	154 (13.8)
Two or more comorbidities	4826 (16.0)	292 (26.1)
Obesity (BMI ≥ 30 kg/m^2^)	1127 (3.7)	42 (3.8)
Diabetes	2170 (7.2)	161 (14.4)
Hypertension	7116 (23.6)	298 (26.7)
Hyperlipidemia	4346 (14.4)	198 (17.7)
Cardiovascular disease	809 (2.7)	135 (12.1)
Cerebrovascular disease	466 (1.5)	145 (13.0)
Chronic kidney disease	509 (1.7)	138 (12.4)
Chronic hepatitis or cirrhosis	425 (1.4)	148 (13.2)
Chronic obstructive pulmonary disease	369 (1.2)	146 (13.1)
Asthma	1175 (3.9)	181 (16.2)
Malignant tumor	717 (2.4)	152 (13.6)
Immunodeficiency^§^	678 (2.3)	140 (12.5)
Engagement in infection prevention measures, n (%)^¶^		
Below average	11,476 (38.1)	671 (60.1)
Above average (more careful)	18,654 (61.9)	446 (39.9)
SARS-CoV-2 vaccination status, n (%)		
No or one time of mRNA vaccination	4329 (14.4)	239 (21.4)
Two or three times of vaccination	25,801 (85.6)	878 (78.6)
Year of infection, n (%)		
Over one year prior to the survey	–	591 (52.9)
Within the preceding year	–	546 (48.9)
Hospitalization due to COVID-19, n (%)		
No	–	616 (55.1)
Yes	–	501 (44.9)
Oxygen administration due to COVID-19, n (%)		
No	–	641 (57.4)
Yes	–	476 (42.6)

*Percentages in all participants (left column)/participants infected with SARS-CoV-2 (right column) (same as below). ^†^21 prefectures that had been declared the State of Emergency more than once. ^‡^Under 18 years old. ^§^Include use of corticosteroids, biologics, and immunosuppressive drugs. ^¶^Percentage of 12 infection prevention behaviors being performed. *n* number (weighted by inverse probability weighting), *SARS-CoV-2* severe acute respiratory syndrome coronavirus 2, *HTPs* heated tobacco products, *METs *metabolic equivalents, *BMI* body mass index, *mRNA* messenger ribonucleic acid, *COVID-19* coronavirus disease 2019.

For the second analysis, we performed multivariable logistic regression analyses to investigate associations between tobacco use status and severe COVID-19 (hospitalization and oxygen administration). Age, sex, comorbidities (as above), and vaccination status were adjusted as covariates. In these analyses, we also adjusted for the year of infection because SARS-CoV-2 strains predominant at the time and vaccination status differed between infection within the preceding year and infection over one year prior to the survey. Those infected within the preceding year were examined based on tobacco use status and vaccination status at the time of the survey, and those infected over one year prior to the survey were examined based on their tobacco use status at one year prior to the survey (since vaccination had started in Japan in February 2021^29^, these respondents were considered unvaccinated).

Using these models, adjusted odds ratios (aORs) and 95% confidence intervals (CIs) were obtained, and values of *p* < 0.05 were considered statistically significant. We also examined the variance inflation factor (VIF) for each covariate and confirmed that covariates were not linear with each other (VIF < 2 was considered to indicate non-linearity).

The population bias due to being an internet survey was adjusted for using inverse probability weighting (IPW). To estimate statistical weightings, we used an existing nationally representative sample from the Comprehensive Survey of Living Conditions of People on Health and Welfare (CSLCPHW)^30^. Two surveys (JASTIS and CSLCPHW) were combined and logistic regression analysis adjusted for background factors such as residence, education, housing tenure, and health status was used to estimate the probability of “being a respondent in an internet survey,” i.e., propensity score. Details on IPW were provided in previous reports^24,26^. The numbers of individuals shown in the text and tables indicate the weighted numbers; unweighted numbers are shown in Supplementary Tables.

All analyses were performed using R (version 4.0.3) and EZR on R commander (version 1.54)^31^.

## Results

A total of 30,130 individuals between 16 and 81 years of age participated in the study, comprising 14,718 men (48.8%) and 15,412 women (51.2%). Of these, 1117 (714 men, 403 women) reported having been infected with SARS-CoV-2. Characteristics of all participants and infected individuals are shown in Table 1. The mean age of all participants was 47.9 years, while that of the infected was 34.9 years, with a bias toward younger age groups. The percentages of current tobacco users at the time of the survey were 24.3% in all and 53.0% among those infected, and current users of HTPs (including dual users) among current tobacco users were 51.3% and 84.5%, respectively.

Among those infected (excluding those infected twice), 525 were infected within the preceding year and 571 were infected over one year prior to the survey (Supplementary Table S3). The percentage of current tobacco users was higher in previously infected individuals than in recently infected ones (73.0% vs. 30.9%), and many individuals were hospitalized (67.3% vs. 18.9%) and received oxygen (66.9% vs. 15.0%) due to COVID-19 in the same group. Among recently infected individuals, 82.5% had been vaccinated against SARS-CoV-2 twice or more.

Results of the first analysis of the association between tobacco use status and SARS-CoV-2 infection among all participants (n = 30,130) are shown in Table 2. Current users of HTPs (aOR = 1.65, 95%CI 1.26–2.15) and current dual users of combustible cigarettes and HTPs (aOR = 4.66, 95%CI 3.89–5.58) were more likely to be infected with SARS-CoV-2 compared to never-users. Former users and current users of combustible cigarettes showed no significant difference in infection rates when compared to never-users. In addition, individuals who were working in some occupations (healthcare worker/remote worker/other worker: aOR = 1.48/1.93/1.35; 95%CI 1.11–1.98/1.42–2.61/1.10–1.65), living in prefectures under alert (many of which were densely populated areas) (aOR = 1.72, 95%CI 1.50–1.99), and frequently interacting with people living separately (aOR = 1.63, 95%CI 1.43–1.86), as well as individuals who were highly physically active (aOR = 1.63, 95%CI 1.43–1.87), were more likely to be infected compared to those who were not. Individuals with two or more comorbidities were more likely to be infected than those without comorbidities (aOR = 2.71, 95%CI 2.26–3.25). SARS-CoV-2 vaccination status was not associated with infection history, but individuals engaging in a high percentage of infection prevention measures were less likely to be infected compared to those with a low percentage (aOR = 0.57, 95%CI 0.50–0.65). A similar analysis was performed using the data for tobacco use/SARS-CoV-2 vaccination status at one year prior to the survey (as mentioned above, no vaccinations had been administered at that time in Japan), yielding similar results (data not shown). In addition, there was no obvious change in tobacco use status at the time of the survey from one or two years prior to the survey (Supplementary Table S4). Adjustment using current status at the time of the survey for risk assessment was therefore considered appropriate.

**Table 2:** Factors associated with SARS-CoV-2 infection among all participants (n = 30,130).

	SARS-CoV-2 infection (n = 1117)			
	n (%)*	Adjusted OR^†^	95%CI	*p*
Age				
≤ 34 years	657 (7.78)	1 (reference)	–	–
35–49 years	289 (3.76)	**0.44**	0.37–0.51	< 0.001
50–64 years	108 (1.62)	**0.21**	0.17–0.27	< 0.001
≥ 65 years	62 (0.85)	**0.14**	0.10–0.19	< 0.001
Sex				
Male	714 (4.85)	1.10	0.95–1.28	0.187
Female	403 (2.61)	1 (reference)	–	–
Tobacco use status				
Never-user	380 (2.34)	1 (reference)	–	–
Former user (combustible cigarettes or HTPs)	145 (2.20)	1.19	0.96–1.46	0.109
Current user of combustible cigarettes	92 (2.58)	1.12	0.88–1.43	0.357
Current user of HTPs	75 (4.81)	**1.65**	1.26–2.15	< 0.001
Current dual user (combustible cigarettes and HTPs)	425 (19.27)	**4.66**	3.89–5.58	< 0.001
Occupation				
Student/unemployed/retiree	157 (1.53)	1 (reference)	–	–
Healthcare worker	94 (4.15)	**1.48**	1.11–1.98	0.008
Remote worker	85 (5.59)	**1.93**	1.42–2.61	< 0.001
Other worker	781 (4.86)	**1.35**	1.10–1.65	0.004
Household annual income				
Below average	545 (3.72)	1 (reference)	–	–
Above average (higher)	374 (4.46)	1.00	0.85–1.16	0.962
Did not know/did not want to answer	198 (2.79)	0.97	0.81–1.16	0.740
Education				
Junior high school/high school	555 (3.55)	1 (reference)	–	–
University/technical school or more	561 (3.86)	0.97	0.85–1.10	0.611
Housing tenure				
Own house	728 (3.21)	1 (reference)	–	–
Rental house	359 (5.20)	1.05	0.89–1.22	0.582
Other (freeloader/rootless)	30 (5.34)	1.33	0.87–2.04	0.191
Living in prefectures under alert^‡^				
No	315 (2.63)	1 (reference)	–	–
Yes	802 (4.42)	**1.72**	1.50–1.99	< 0.001
Household members other than the participant				
None (living alone)	249 (4.89)	1 (reference)	–	–
Adult(s) only	425 (2.55)	0.86	0.71–1.04	0.122
Living with a child (children)^§^	443 (5.28)	1.20	0.99–1.46	0.067
Interacting with people living separately at least once a week				
No	491 (2.52)	1 (reference)	–	–
Yes	625 (5.85)	**1.63**	1.43–1.86	< 0.001
Total METs per day				
Below average	607 (2.70)	1 (reference)	–	–
Above average (more active)	510 (6.67)	**1.63**	1.43–1.87	< 0.001
Alcohol consumption				
Never drinker	394 (3.01)	1 (reference)	–	–
Ever drinker	723 (4.24)	1.08	0.94–1.25	0.257
Comorbidities^¶^				
None	671 (3.58)	1 (reference)	–	–
One comorbidity	154 (2.35)	1.16	0.96–1.41	0.120
Two or more comorbidities	292 (6.05)	**2.71**	2.26–3.25	< 0.001
Engagement in infection prevention measures^||^				
Below average	671 (5.85)	1 (reference)	–	–
Above average (more careful)	446 (2.39)	**0.57**	0.50–0.65	< 0.001
SARS-CoV-2 vaccination status				
No or one time of mRNA vaccination	239 (5.52)	1 (reference)	–	–
Two or three times of vaccination	878 (3.40)	0.90	0.76–1.06	0.212

*Percentages of infected individuals in each category. ^†^Estimated using multivariable logistic regression modeling with adjustment for all listed variables. Values of *p* < 0.05 were considered statistically significant and corresponding adjusted ORs are shown in bold. ^‡^21 prefectures that had been declared the State of Emergency more than once. ^§^Under 18 years old. ^¶^12 diseases listed in Table 1. ^||^Percentage of 12 infection prevention behaviors being performed. *SARS-CoV-2* severe acute respiratory syndrome coronavirus 2, *n* number (weighted by inverse probability weighting), *OR* odds ratio, *CI* confidence interval, *HTPs* heated tobacco products, *METs* metabolic equivalents, *mRNA* messenger ribonucleic acid.

Results of the second analysis of the associations between tobacco use status and severe COVID-19 (hospitalization and oxygen administration) among those infected (n = 1097) are shown in Table 3. Of the infected respondents, 483 (44.0%) were hospitalized and 461 (42.0%) were administered oxygen. Current dual users of combustible cigarettes and HTPs compared to never-users (aOR = 3.17, 95%CI 2.11–4.77) and individuals who had two or more comorbidities compared to those without comorbidities (aOR = 1.46, 95%CI 1.01–2.11) were more likely to be hospitalized due to COVID-19. All tobacco users (including former users) were more likely to be administered oxygen compared to never-users (former user/current user of combustible cigarettes/current user of HTPs: aOR = 1.88/3.17/1.90, 95%CI 1.11–3.19/1.77–5.67/1.01–3.59), and dual users required oxygen administration the most at the time of infection (aOR = 4.15, 95%CI 2.70–6.36). Individuals with multiple comorbidities were also more likely to be administered oxygen than those without comorbidities (aOR = 1.86, 95%CI 1.27–2.72). Individuals vaccinated twice or more had almost half the aORs for hospitalization and oxygen administration compared to those who received no or only one dose of vaccination (hospitalization/oxygen administration: aOR = 0.57/0.49, 95%CI 0.33–0.98/0.27–0.87) even after taking into account differences in severity rate by year of infection. Neither age group nor sex were associated with severe disease in this population.

**Table 3:** Factors associated with hospitalization and oxygen administration due to COVID-19 among participants infected with SARS-CoV-2 once (n = 1097).

	Hospitalization due to COVID-19 (n = 483)				Oxygen administration due to COVID-19 (n = 461)			
	n (%)*	Adjusted OR^†^	95%CI	*p*	n (%)*	Adjusted OR^†^	95%CI	*p*
Age								
≤ 34 years	292 (45.6)	1 (reference)	–	–	290 (45.2)	1 (reference)	–	–
35–49 years	128 (44.9)	1.17	0.83–1.66	0.371	117 (41.1)	0.90	0.63–1.30	0.583
50–64 years	41 (38.0)	1.63	0.97–2.73	0.065	34 (31.5)	1.12	0.63–1.98	0.704
≥ 65 years	21 (33.9)	1.23	0.64–2.37	0.540	20 (32.3)	1.33	0.66–2.67	0.419
Sex								
Male	361 (52.0)	1.09	0.78–1.53	0.612	357 (51.4)	1.18	0.82–1.69	0.377
Female	123 (30.5)	1 (reference)	–	–	103 (25.6)	1 (reference)	–	–
Tobacco use status^‡^								
Never-user	88 (23.3)	1 (reference)	–	–	63 (16.7)	1 (reference)	–	–
Former user (combustible cigarettes or HTPs)	45 (31.7)	1.30	0.79–2.11	0.301	42 (29.6)	**1.88**	1.11–3.19	0.019
Current user of combustible cigarettes	35 (37.6)	1.22	0.70–2.13	0.484	43 (46.2)	**3.17**	1.77–5.67	< 0.001
Current user of HTPs	26 (37.7)	1.27	0.70–2.32	0.438	25 (36.2)	**1.90**	1.01–3.59	0.048
Current dual user (combustible cigarettes and HTPs)	289 (69.5)	**3.17**	2.11–4.77	< 0.001	288 (69.2)	**4.15**	2.70–6.36	< 0.001
Comorbidities^§^								
None	233 (35.0)	1 (reference)	–	–	217 (32.6)	1 (reference)	–	–
One comorbidity	63 (42.3)	1.27	0.82–1.97	0.288	47 (31.5)	0.79	0.50–1.27	0.333
Two or more comorbidities	188 (66.4)	**1.46**	1.01–2.11	0.044	197 (69.6)	**1.86**	1.27–2.72	0.001
SARS-CoV-2 vaccination status^‡^								
No or one time of mRNA vaccination	410 (61.7)	1 (reference)	–	–	405 (61.0)	1 (reference)	–	–
Two or three times of vaccination	73 (16.9)	**0.57**	0.33–0.98	0.042	55 (12.7)	**0.49**	0.27–0.87	0.015
Year of infection								
Over one year prior to the survey	384 (67.3)	1 (reference)	–	–	382 (66.9)	1 (reference)	–	–
Within the preceding year	99 (18.9)	**0.26**	0.16–0.44	< 0.001	79 (15.0)	**0.25**	0.14–0.42	< 0.001

*Percentages of individuals who hospitalized (left column)/administered oxygen (right column) among those infected once in each category. ^†^Estimated using multivariable logistic regression modeling with adjustment for all listed variables. Values of *p* < 0.05 were considered statistically significant and corresponding adjusted ORs are shown in bold. ^‡^Reflected the status at the time of the survey for infection within the preceding year and the status at one year prior to the survey for infection over one year prior to the survey. ^§^12 diseases listed in Table 1. *COVID-19* coronavirus disease 2019, *n* number (weighted by inverse probability weighting), *OR* odds ratio, *CI* confidence interval, *HTPs* heated tobacco products, *SARS-CoV-2* severe acute respiratory syndrome coronavirus 2, *mRNA* messenger ribonucleic acid.

The results of the second analysis adjusted for each comorbidity are shown in Supplementary Table S5, and the results for the unweighted population are shown in Supplementary Tables S6, S7, and S8.

## Discussion

We examined the association of tobacco use including HTPs with SARS-CoV-2 infection and severe COVID-19 using data from a large-scale questionnaire. Logistic regression modeling revealed that use of HTPs alone or in combination with combustible cigarettes was associated with higher rates of SARS-CoV-2 infection and severe COVID-19 than never-use, and both rates were markedly higher in the combination group than in the other tobacco status groups. To the best of our knowledge, this represents the first report to examine associations between use of tobacco including HTPs and severe COVID-19.

While many COVID-19 patients were asymptomatic and therefore undiagnosed^32^, one report suggested that use of e-cigarettes had increased the percentage of symptomatic patients at the time of SARS-CoV-2 infection^33^. In the present study, many users of HTPs were diagnosed as infected, and this may be due to an increase in symptomatic patients among users of HTPs. In addition, Japan had no lockdown period during the COVID-19 outbreak, and Japanese citizens were only asked to refrain from going out through the declaration of the State of Emergency when the number of infected people increased (Supplementary Table S1). In this study population, users of HTPs were more physically active than non-users (average 47.1 vs. 38.8 METs per day; data not shown). Although we adjusted for confounding factors such as occupation and interaction with people living separately, high activity with frequent outings may have contributed to the increase in infection. Meanwhile, engagement in infection prevention measures was higher among current users of HTPs than among others, including users of combustible cigarettes (average 90.6% vs. 83.7%; data not shown), suggesting that health awareness may have increased the number of those tested for SARS-CoV-2.

Regarding history of hospitalization, no significant associations were evident among tobacco users other than dual users. In Japan, there was a period during which all COVID-19 patients were hospitalized at the beginning of the outbreak, and thus hospitalization may not necessarily reflect disease severity. Because many tobacco users were among those infected over one year prior to the survey (Supplementary Table S3), corresponding to the above period, the association with hospitalization may not have been significant, unlike with oxygen administration.

Use of combustible cigarettes is generally considered a risk factor for severe COVID-19, including increased risk of respiratory failure requiring mechanical ventilation, and death^15–17^, whereas few reports have described the association between new tobacco products and COVID-19. Similar to the abovementioned papers, current use of combustible cigarettes was associated with severe COVID-19 (i.e., oxygen administration) in the present study. As for HTPs, current single users displayed an aOR for oxygen administration almost equal to or slightly higher than that in former tobacco users. Since 86.0% of single users of HTPs had a history of using combustible cigarettes, the inference was that they were affected by past use of combustible cigarettes, like the majority of former tobacco users. In addition, one report from Japan noted a decrease in the immunoglobulin G antibody titer after SARS-CoV-2 vaccination in current users of tobacco, including HTPs^34^, which may have been associated with severe disease in those infected within the preceding year in the present study. Interestingly, however, dual users showed a markedly higher aOR for oxygen administration than those in other tobacco status groups. Dual users may have used combustible cigarettes in situations where its use was possible and have used HTPs where it was not^35^, and may have had contact with others without a mask in areas designated for cigarette smoking and other spaces. Wearing a mask is hypothesized to reduce the amount of inhaled virus and make COVID-19 less severe^36,37^, and so the amount of viral exposure might have been related to disease severity. However, if dual use itself increases the risk of severe disease, the mechanism awaits further study.

Infected respondents in this study skewed younger than in previous studies of hospitalized patients^20,27^. With regard to severity, older age groups had higher aORs than younger age group, but unlike previous reports^16,27^, no significant association with age was seen. This may be because elderly individuals at higher risk of severe disease than healthy ones, such as those already hospitalized or in nursing homes, were unable to participate. Similarly, no association between sex and COVID-19 severity was identified; this may be because tobacco use and other factors were adjusted for as covariates in the analysis. The tobacco use rate was higher among males than among females (Supplementary Table S9), and males had a significantly higher odds ratio for severe disease than females in univariable analysis (Supplementary Table S10).

Presence of one comorbidity was also not associated with severity. In this study, comorbidities were selected from those associated with COVID-19 severity in previous reports^16,27^. Diseases among individuals with one comorbidity were hypertension in 46.6% and hyperlipidemia in 24.3%, neither of which are listed by the United States Centers for Disease Control and Prevention as risk factors for severe COVID-19^38^ because of inconsistent conclusions among reports. We thus consider that having only a single comorbidity was not associated with severe disease.

SARS-CoV-2 vaccination, once adjusted for engagement in infection prevention measures, was not associated with infection history. A past report has indicated that post-vaccination breakthrough infections were common, particularly with mutant strains of SARS-CoV-2, but vaccination prevented severe disease^39^. Similarly, in this study, even after adjusting for the year of infection (i.e., the viral strains predominant at the time), individuals vaccinated twice or more were less likely to develop severe disease compared to those who received no or only one dose of vaccination.

### Limitations

This study had several limitations that should be kept in mind.

First, this was a cross-sectional study that only compared tobacco use status and other background characteristics with history of SARS-CoV-2 infection and severe COVID-19, and so did not examine causal relationships.

Second, administration as an internet survey introduced bias in participant selection. The obtained data were weighted using IPW, but because this was a study of a sample from the general population, not hospitalized patients, severely ill or deceased COVID-19 patients (particularly elderly individuals) would not have had access to this survey. This may help explain the bias toward younger age among the infected and why older age was not associated with severe disease in this study.

Third, this was a questionnaire-based survey, and the diagnosis of COVID-19 was self-reported. However, because of the great interest in COVID-19 at this time, while asymptomatic individuals may not have been diagnosed, at least symptomatic ones were likely to have been examined or tested for the virus and would have known their results. History of severe disease was also self-reported, and no questions investigated interventions beyond oxygen administration (e.g., use of ventilators or extracorporeal membrane oxygenation). In this regard, since we defined severe disease as hospitalization or oxygen administration, which were easily understood by the general population, the patients themselves were likely to have known whether they had been in these situations.

Fourth, actual dates of infection were not known and the exact circumstances at the time of infection may not have been reflected in the analyses. For infection, similar results were obtained by adjusting for tobacco use/vaccination status at the time of the survey and at one year prior to the survey. For severe disease, results were adjusted by the status of the year of infection, which likely reflected the actual circumstances. We therefore believe that the possibility of reverse causation such as “tobacco cessation/vaccination because of infection” was eliminated to the extent possible.

Fifth, the amount and duration of tobacco use was not considered, and users of HTPs may have been using combustible cigarettes for longer than HTPs, regardless of their current use of combustible cigarettes. Nevertheless, dual users displayed the highest aORs for infection and severe disease, suggesting that some effect may have been introduced with combined use.

Finally, we could not exclude the remaining confounding factors related to infection and severe disease, including unknown factors.

## Conclusion

In a sample of the Japanese study population in 2022, current tobacco users comprised 24.3%, of which 21.2% were single users of HTPs and 30.1% were dual users of combustible cigarettes and HTPs. Compared to never-users, current users of HTPs (including dual users) were more likely to be infected with SARS-CoV-2, and current or former users of combustible cigarettes and/or HTPs were more likely to require oxygen administration due to COVID-19. Our results are useful in considering the safety of tobacco products use during the ongoing COVID-19 pandemic in the context of the current situation in Japan and, by extension, in other regions where the use of HTPs is expanding.

## Supplementary Information

Supplementary Information.

## Data Availability

The data used in this study are not available in a public repository because they contain personally identifiable or potentially sensitive participants’ information, but de-identified data are available from Takahiro Tabuchi (Cancer Control Center, Osaka International Cancer Institute, Osaka, Japan; E-mail: tabuchitak@gmail.com) to qualified researchers on reasonable request.

## References

[1] Pepper JK, Brewer NT (2014). Electronic nicotine delivery system (electronic cigarette) awareness, use, reactions and beliefs: A systematic review. Tob Control..

[2] Uchiyama S (2018). Simple determination of gaseous and particulate compounds generated from heated tobacco products. Chem. Res. Toxicol..

[3] Tabuchi T (2018). Heat-not-burn tobacco product use in Japan: Its prevalence, predictors and perceived symptoms from exposure to secondhand heat-not-burn tobacco aerosol. Tob Control..

[4] Okawa S, Tabuchi T, Miyashiro I (2020). Who uses e-cigarettes and why? e-cigarette use among older adolescents and young adults in Japan: JASTIS study. J Psychoactive Drugs..

[5] Tobacco Institute of Japan. Data concerning tobacco. Available online at: https://www.tioj.or.jp/data/ Accessed 22 June 2022.

[6] Kondo T, Nakano Y, Adachi S, Murohara T (2019). Effects of tobacco smoking on cardiovascular disease. Circ. J..

[7] U.S. National Cancer Institute Tobacco. Cancer causes and prevention. Risk factors. Tobacco. Available online at: https://www.cancer.gov/about-cancer/causes-prevention/risk/tobacco/ Accessed 17 July 2022 (2017).

[8] Hwang JH, Ryu DH, Park SW (2019). Heated tobacco products: Cigarette complements, not substitutes. Drug Alcohol Depend..

[9] Auer R, Concha-Lozano N, Jacot-Sadowski I, Cornuz J, Berthet A (2017). Heat-not-burn tobacco cigarettes: Smoke by any other name. JAMA Intern. Med..

[10] Aokage T (2019). Heat-not-burn cigarettes induce fulminant acute eosinophilic pneumonia requiring extracorporeal membrane oxygenation. Respir. Med. Case Rep..

[11] Tajiri T (2020). Acute eosinophilic pneumonia induced by switching from conventional cigarette smoking to heated tobacco product smoking. Intern. Med..

[12] Hoffmann M (2020). SARS-CoV-2 cell entry depends on ACE2 and TMPRSS2 and is blocked by a clinically proven protease inhibitor. Cell.

[13] Brake SJ (2020). Smoking upregulates angiotensin-converting enzyme-2 receptor: A potential adhesion site for novel coronavirus SARS-CoV-2 (Covid-19). J. Clin. Med..

[14] Oakes JM, Fuchs RM, Gardner JD, Lazartigues E, Yue X (2018). Nicotine and the renin-angiotensin system. Am. J. Physiol. Regul. Integr. Comp. Physiol..

[15] Zhang H (2021). Association of smoking history with severe and critical outcomes in COVID-19 patients: A systemic review and meta-analysis. Eur. J. Integr. Med..

[16] Zheng Z (2020). Risk factors of critical and mortal COVID-19 cases: A systematic literature review and meta-analysis. J. Infect..

[17] Patanavanich R, Glantz SA (2020). Smoking is associated with COVID-19 progression: A meta-analysis. Nicotine Tob. Res..

[18] Farsalinos K (2021). Smoking prevalence among hospitalized COVID-19 patients and its association with disease severity and mortality: An expanded re-analysis of a recent publication. Harm. Reduct. J..

[19] Williamson EJ (2020). Factors associated with COVID-19-related death using OpenSAFELY. Nature.

[20] Matsushita Y (2022). Smoking and severe illness in hospitalized COVID-19 patients in Japan. Int. J. Epidemiol..

[21] Ho LLK (2020). Impact of COVID-19 on the Hong Kong Youth Quitline service and quitting behaviors of its users. Int. J. Environ. Res. Public Health.

[22] Kim J, Lee S (2022). Impact of the COVID-19 pandemic on tobacco sales and national smoking cessation services in Korea. Int. J. Environ. Res. Public Health..

[23] Gallus S (2022). Use of electronic cigarettes and heated tobacco products during the Covid-19 pandemic. Sci. Rep..

[24] Tabuchi T, Shinozaki T, Kunugita N, Nakamura M, Tsuji I (2019). Study profile: the Japan "society and new tobacco" internet survey (JASTIS): A longitudinal internet cohort study of heat-not-burn tobacco products, electronic cigarettes, and conventional tobacco products in Japan. J. Epidemiol..

[25] Amato MS, Boyle RG, Levy D (2016). How to define e-cigarette prevalence? Finding clues in the use frequency distribution. Tob. Control.

[26] Tabuchi T (2016). Awareness and use of electronic cigarettes and heat-not-burn tobacco products in Japan. Addiction.

[27] Terada M (2021). Risk factors for severity on admission and the disease progression during hospitalisation in a large cohort of patients with COVID-19 in Japan. BMJ Open.

[28] Gotanda H, Miyawaki A, Tabuchi T, Tsugawa Y (2021). Association between trust in government and practice of preventive measures during the COVID-19 pandemic in Japan. J. Gen. Intern. Med..

[29] Japan Ministry of Health, Labour and Welfare. SARS-CoV-2 vaccination results. Available online at: https://www.mhlw.go.jp/stf/seisakunitsuite/bunya/vaccine_sesshujisseki.html Accessed 27 July 2022 (2021).

[30] Japan Ministry of Health, Labour and Welfare. Overview of the 2016 Comprehensive Survey of Living Condition of the People on Health and Welfare. Available online at: https://www.mhlw.go.jp/toukei/saikin/hw/k-tyosa/k-tyosa16/index.html Accessed 19 December 2022 (2016).

[31] Kanda Y (2013). Investigation of the freely available easy-to-use software 'EZR' for medical statistics. Bone Marrow Transpl..

[32] Gao Z (2021). A systematic review of asymptomatic infections with COVID-19. J. Microbiol. Immunol. Infect..

[33] McFadden DD (2022). Symptoms COVID 19 positive vapers compared to COVID 19 positive non-vapers. J. Prim. Care Commun. Health.

[34] Yamamoto S (2022). Use of heated tobacco products, moderate alcohol drinking, and anti-SARS-CoV-2 IgG antibody titers after BNT162b2 vaccination among Japanese healthcare workers. Prev. Med..

[35] Kiyohara K, Tabuchi T (2022). Use of heated tobacco products in smoke-free locations in Japan: The JASTIS 2019 study. Tob. Control..

[36] Chan JF (2020). Surgical mask partition reduces the risk of noncontact transmission in a golden Syrian hamster model for coronavirus disease 2019 (COVID-19). Clin. Infect. Dis..

[37] Levine Z, Earn DJD (2022). Face masking and COVID-19: Potential effects of variolation on transmission dynamics. J. R. Soc. Interface..

[38] Centers for Disease Control and Prevention. At increased risk for severe illness. People with certain medical conditions. Available online at: https://www.cdc.gov/coronavirus/2019-ncov/need-extra-precautions/people-with-medical-conditions.html Accessed 22 July 2022 (2022).

[39] Cohn BA, Cirillo PM, Murphy CC, Krigbaum NY, Wallace AW (2022). SARS-CoV-2 vaccine protection and deaths among US veterans during 2021. Science.

