# Supplementary material for: Association of combustible cigarettes and heated tobacco products use with SARS-CoV-2 infection and severe COVID-19 in Japan: a JASTIS 2022 cross-sectional study
Source: Sci Rep. 2023 Feb 2;13:1120. doi: 10.1038/s41598-023-28006-3 (PMC9894839; doi:10.1038/s41598-023-28006-3)
Supplement: Supplementary file 1 — Supplementary Information. [file 41598_2023_28006_MOESM1_ESM.pdf]

Supplementary Information

Title

Association of combustible cigarettes and heated tobacco products use with SARS-CoV-2 infection and COVID-19 severity in Japan: a JASTIS 2022 cross-sectional study

Authors

Misako Nishimura, Kazuhisa Asai, Takahiro Tabuchi, Erika Toyokura, Takahiro Kawai, Atsushi Miyamoto, Tetsuya Watanabe, Tomoya Kawaguchi

List

Supplementary Note. Definition of incorrect or unnatural answers; Definition of variables (Additional).

Supplementary Table S1. Prefectures under alert.

Supplementary Table S2. Infection prevention measures.

Supplementary Table S3. Characteristics of participants infected with SARS-CoV-2 once.

Supplementary Table S4. Tobacco use status at the time of the survey, one year prior, and two years prior to the survey.

Supplementary Table S5. Factors associated with hospitalization and oxygen administration due to COVID-19 among participants infected with SARS-CoV-2 once (Adjusted for each comorbidity).

Supplementary Table S6. Characteristics of participants (Unweighted).

Supplementary Table S7. Factors associated with SARS-CoV-2 infection among all participants (Unweighted).

Supplementary Table S8. Factors associated with hospitalization and oxygen administration due to COVID-19 among participants infected with SARS-CoV-2 once (Unweighted).

Supplementary Table S9. Tobacco use status of male and female.

Supplementary Table S10. Factors associated with hospitalization and oxygen administration due to COVID-19 among participants infected with SARS-CoV-2 once (Univariable analysis).

Supplementary Note.

Definition of incorrect or unnatural answers.

Those meeting any of the following three criteria were considered for exclusion.
1) Those who selected other than the second in the question of “please select the second option from the bottom out of five options (A, B, C, D, and E).”
2) Those who selected “yes” to all drug use (do you use the following drugs? alcohol, strong chu-hi with an alcohol content of 9% or more, sleeping pills / anti-anxiety medications, morphine, thinner, marijuana, dangerous drugs [e.g., outlawed herbs, magic mushrooms, etc.], and cocaine / heroin [eight items]).
3) Those who selected “yes” to comorbidity of all diseases (do you have the following diseases? hypertension, diabetes, asthma, atopic dermatitis, allergic rhinitis, angina pectoris / myocardial infarction, stroke [cerebral infarction or cerebral hemorrhage], cancer / malignant tumor, and chronic pain [nine items]).

Definition of variables (Additional).

Remote workers: those who were working remotely for at least half of the week at the time of the survey excluding unemployed and healthcare workers.
Household annual income: options were “none, less than 500,000 yen, 500,000 yen to 1 million yen, (similar items in 1 to 2 million yen increments until 20 million yen), more than 20 million yen, did not know, and did not want to answer”; the median value was used for those with a wide range, 30 million yen for “more than 20 million yen” and missing values for the last two, then the average (weighted mean) of those who answered the amount was calculated. The last two were placed in a separate category.
Prefectures under alert: prefectures in Japan that had been declared the State of Emergency more than once (listed in Supplementary Table S7)[1].
Metabolic equivalents (METs): the amount of activity per hour was defined as “physical labor and intense sports = 4.5 METs, sitting = 1.5 METs, walking or standing = 2 METs, and other activity = 1.5 METs”; options for hours per day for each item were “none, less than 0.5 hours, about 0.5 hours, 1 hour, 2 hours, 3 hours, 4 to 5 hours, (similar items in 1 hour increments until 12 hours), more than 12 hours, and unknown”; “less than 0.5 hours” was considered 15 minutes, “more than 12 hours” was considered 12 hours, median value was used for those with a wide range, and the average time of others was used for “unknown”, and then the average (weighted mean) of all values was calculated[2].
Engagement in infection prevention measures: for each of the 12 infection prevention behaviors considered desirable in Japan (Supplementary Table S10), the percentages of items that were “always done” or “sometimes done” were calculated, and the average (weighted mean) was obtained (options were “always done, sometimes done, seldom done, and never done”)[3].

Prefectures declared the State of Emergency in Japan	
1st (2020.4.7–2020.5.25)	All 47 prefectures
2nd (2021.1.8–2021.3.21)	11 prefectures: Tochigi, Saitama, Chiba, Tokyo, Kanagawa, Gifu, Aichi, Kyoto, Osaka, Hyogo, and Fukuoka
3rd (2021.4.25–2021.9.30)	21 prefectures: Hokkaido, Miyagi, Ibaraki, Tochigi, Gunma, Saitama, Chiba, Tokyo, Kanagawa, Gifu, Shizuoka, Aichi, Mie, Shiga, Kyoto, Osaka, Hyogo, Okayama, Hiroshima, Fukuoka, and Okinawa

Supplementary Table S1. Prefectures under alert. 21 prefectures in Japan that had been declared the State of Emergency (request for cooperation in infection prevention measures by the government, including voluntary refraining from outings and restricting the use of facilities) more than once[1] were defined as prefectures under alert.

Infection prevention behaviors preferred in Japan	
1.	Disinfected hands and fingers with rubbing alcohol
2.	Washed hands for more than 15 seconds with bar soap or hand soap
3.	Gargled when returning home
4.	Did not touch eyes, nose, or mouth with unwashed hands
5.	Wore a mask where there were people
6.	Refrained from traveling
7.	Refrained from unnecessary and unurgent going out and business trips
8.	Did not talk or speak at close range (within 1 meter)
9.	Took social distance (more than 2 meters) from others
10.	Did not meet someone who was likely to get infected
11.	Did not go to a crowded place
12.	Refrained from eating in a restaurant

Supplementary Table S2. Infection prevention measures. Percentage of items done was calculated for these 12 items as in Supplementary Note.

	Infected within the preceding year (n=525)	Infected over one year prior to the survey (n=571)
**Age, mean (years)**	37.0	33.3
**Tobacco use status, n (%)*†**		
Never-user	268 (51.0)	108 (18.9)
Former user (combustible cigarettes or HTPs)	96 (18.3)	46 (8.1)
Current user of combustible cigarettes	45 (8.6)	48 (8.4)
Current user of HTPs	33 (6.3)	36 (6.3)
Current dual user (combustible cigarettes and HTPs)	84 (16.0)	333 (58.3)
**Comorbidities, n (%)‡**		
None	375 (71.4)	290 (50.8)
One comorbidity	83 (15.8)	66 (11.6)
Two or more comorbidities	67 (12.8)	216 (37.8)
**SARS-CoV-2 vaccination status, n (%)†**		
No or one time of mRNA vaccination	93 (17.7)	571 (100)
Two or three times of vaccination	433 (82.5)	0 (0)
**Hospitalization due to COVID-19, n (%)**		
No	426 (81.1)	187 (32.7)
Yes	99 (18.9)	384 (67.3)
**Oxygen administration due to COVID-19, n (%)**		
No	447 (85.1)	189 (33.1)
Yes	79 (15.0)	382 (66.9)

Supplementary Table S3. Characteristics of participants infected with SARS-CoV-2 once. *Percentages in those infected within the preceding year (left column) / over one year prior to the survey (right column) (same as below). †Reflected the status at the time of the survey for infection within the preceding year and the status at one year prior to the survey for infection over one year prior to the survey. ‡12 diseases listed in Table 1. n: number (weighted by inverse probability weighting), HTPs: heated tobacco products, SARS-CoV-2: severe acute respiratory syndrome coronavirus 2, mRNA: messenger ribonucleic acid, COVID-19: coronavirus disease 2019.

	All participants (n=30,130)			Participants infected with SARS-CoV-2 (n=1,117)		
	At the time of the survey	One year prior to the survey	Two years prior to the survey	At the time of the survey	One year prior to the survey	Two years prior to the survey
**Tobacco use status, n (%)***						
Never-user	16210 (53.8)	16210 (53.8)	16210 (53.8)	380 (34.0)	380 (34.0)	380 (34.0)
Former user (combustible cigarettes or HTPs)	6584 (21.9)	6275 (20.8)	6038 (20.0)	145 (13.0)	138 (12.4)	133 (11.9)
Current user of combustible cigarettes	3571 (11.9)	3684 (12.2)	3751 (12.4)	92 (8.2)	91 (8.1)	93 (8.3)
Current user of HTPs	1558 (5.2)	1488 (4.9)	1436 (4.8)	75 (6.7)	65 (5.8)	62 (5.6)
Current dual user (combustible cigarettes and HTPs)	2206 (7.3)	2473 (8.2)	2694 (8.9)	425 (38.0)	443 (39.7)	449 (40.2)

Supplementary Table S4. Tobacco use status at the time of the survey, one year prior, and two years prior to the survey. *Percentages in all participants (left three columns) / participants infected with SARS-CoV-2 (right three columns). n: number (weighted by inverse probability weighting), SARS-CoV-2: severe acute respiratory syndrome coronavirus 2, HTPs: heated tobacco products.

	Hospitalization due to COVID-19 (n=483)				Oxygen administration due to COVID-19 (n=461)			
	**n (%)[*]**	**Adjusted OR[†]**	**95%CI**	***p***	**n (%)[*]**	**Adjusted OR[†]**	**95%CI**	***p***
**Age**								
≤ 34 years	292 (45.6)	1 (reference)	–	–	290 (45.2)	1 (reference)	–	–
35–49 years	128 (44.9)	1.16	0.81–1.66	0.410	117 (41.1)	0.81	0.56–1.18	0.270
50–64 years	41 (38.0)	**1.73**	1.01–2.95	0.046	34 (31.5)	1.25	0.70–2.23	0.455
≥ 65 years	21 (33.9)	1.25	0.63–2.47	0.519	20 (32.3)	1.59	0.78–3.23	0.201
**Sex**								
Male	361 (52.0)	1.12	0.79–1.60	0.518	357 (51.4)	1.44	0.99–2.08	0.056
Female	123 (30.5)	1 (reference)	–	–	103 (25.6)	1 (reference)	–	–
**Tobacco use status[‡]**								
Never-user	88 (23.3)	1 (reference)	–	–	63 (16.7)	1 (reference)	–	–
Former user (combustible cigarettes or HTPs)	45 (31.7)	1.28	0.77–2.10	0.340	42 (29.6)	**1.90**	1.12–3.23	0.018
Current user of combustible cigarettes	35 (37.6)	1.32	0.75–2.32	0.338	43 (46.2)	**3.29**	1.83–5.93	<0.001
Current user of HTPs	26 (37.7)	1.33	0.71–2.46	0.372	25 (36.2)	**2.00**	1.06–3.77	0.033
Current dual user (combustible cigarettes and HTPs)	289 (69.5)	**3.40**	2.22–5.19	<0.001	288 (69.2)	**3.57**	2.30–5.54	<0.001
**Comorbidities**								
Obesity (BMI ≥ $30 kg/m^2$)[§]	21 (51.2)	1.01	0.47–2.16	0.990	23 (56.1)	1.70	0.77–3.72	0.189
Diabetes	87 (56.5)	**0.34**	0.19–0.59	<0.001	96 (62.3)	**0.56**	0.31–0.98	0.044
Hypertension	178 (62.0)	**1.66**	1.07–2.56	0.024	173 (60.3)	1.01	0.64–1.60	0.968
Hyperlipidemia	119 (62.3)	1.03	0.62–1.73	0.903	120 (62.8)	0.97	0.57–1.67	0.922
Cardiovascular disease	102 (76.1)	**2.38**	1.16–4.85	0.017	106 (79.1)	1.71	0.83–3.54	0.147
Cerebrovascular disease	110 (79.7)	**2.29**	1.12–4.66	0.023	114 (82.6)	**2.54**	1.19–5.43	0.016
Chronic kidney disease	89 (67.9)	0.70	0.34–1.46	0.339	96 (73.3)	1.01	0.48–2.09	0.983
Chronic hepatitis or cirrhosis	104 (73.2)	1.84	0.85–3.97	0.122	106 (74.6)	0.55	0.24–1.24	0.148
Chronic obstructive pulmonary disease	102 (70.8)	**0.39**	0.17–0.89	0.024	113 (78.5)	1.75	0.78–3.93	0.178
Asthma	117 (67.2)	0.86	0.46–1.62	0.648	122 (70.1)	0.75	0.39–1.47	0.407
Malignant tumor	103 (69.1)	**0.46**	0.23–0.92	0.029	124 (83.2)	**3.43**	1.69–6.97	<0.001
Immunodeficiency[¶]	102 (76.7)	**2.60**	1.38–4.88	0.003	91 (68.4)	0.51	0.25–1.06	0.072
**SARS-CoV-2 vaccination status[‡]**								
No or one time of mRNA vaccination	410 (61.7)	1 (reference)	–	–	405 (61.0)	1 (reference)	–	–
Two or three times of vaccination	73 (16.9)	0.59	0.34–1.04	0.067	55 (12.7)	**0.49**	0.27–0.89	0.018
**Year of infection**								
Over one year prior to the survey	384 (67.3)	1 (reference)	–	–	382 (66.9)	1 (reference)	–	–
Within the preceding year	99 (18.9)	**0.23**	0.14–0.39	<0.001	79 (15.0)	**0.24**	0.14–0.41	<0.001

Supplementary Table S5. Factors associated with hospitalization and oxygen administration due to COVID-19 among participants infected with SARS-CoV-2 once (n=1,097) (Adjusted for each comorbidity). [*]Percentages of individuals who hospitalized (left column) / administered oxygen (right column) among those infected once in each category. [†]Estimated using multivariable logistic regression modeling with adjustment for all listed variables. Values of $p < 0.05$ were considered statistically significant and corresponding adjusted ORs are shown in bold. [‡]Reflected the status at the time of the survey for infection within the preceding year and the status at one year prior to the survey for infection over one year prior to the survey. [§]References are not having each comorbidity (same as the diseases below). [¶]Include use of corticosteroids, biologics, and immunosuppressive drugs. COVID-19: coronavirus disease 2019, n: number (weighted by inverse probability weighting), OR: odds ratio, 95%CI: 95% confidence interval, HTPs: heated tobacco products, BMI: body mass index, SARS-CoV-2: severe acute respiratory syndrome coronavirus 2, mRNA: messenger ribonucleic acid.

	All participants (n=30,130)	Participants infected with SARS-CoV-2 (n=1,184)
**Age, n (%)***		
≤ 34 years	8620 (28.6)	677 (57.2)
35–49 years	7526 (25.0)	317 (26.8)
50–64 years	6810 (22.6)	127 (10.7)
≥ 65 years	7174 (23.8)	63 (5.3)
**Sex, n (%)**		
Male	14718 (48.8)	729 (61.6)
Female	15412 (51.2)	455 (38.4)
**Tobacco use status, n (%)**		
Never-user	17104 (56.8)	471 (39.8)
Former user (combustible cigarettes or HTPs)	6494 (21.6)	180 (15.2)
Current user of combustible cigarettes	3241 (10.8)	86 (7.3)
Current user of HTPs	1333 (4.4)	91 (7.7)
Current dual user (combustible cigarettes and HTPs)	1958 (6.5)	356 (30.1)
**Occupation, n (%)**		
Student / unemployed / retiree	9841 (32.7)	174 (14.7)
Healthcare worker	2443 (8.1)	112 (9.5)
Remote worker	2220 (7.4)	99 (8.4)
Other worker	15626 (51.9)	799 (67.5)
**Household annual income, n (%)**		
Below average	14466 (48.0)	556 (47.0)
Above average (higher)	9369 (31.1)	452 (38.2)
Did not know / did not want to answer	6295 (20.9)	176 (14.9)
**Education, n (%)**		
Junior high school / high school	8249 (27.4)	288 (24.3)
University / technical school or more	21881 (72.6)	896 (75.7)
**Housing tenure, n (%)**		
Own house	20357 (67.6)	640 (54.1)
Rental house	9175 (30.5)	502 (42.4)
Other (freeloader / rootless)	598 (2.0)	42 (3.5)
**Living in prefectures under alert, n (%)†**		
No	6471 (21.5)	142 (12.0)
Yes	23659 (78.5)	1042 (88.0)
**Household members other than the participant, n (%)**		
None (living alone)	6764 (22.4)	329 (27.8)
Adult(s) only	16020 (53.2)	427 (36.1)
Living with a child (children)‡	7346 (24.4)	428 (36.1)
**Interacting with people living separately at least once a week, n (%)**		
No	19799 (65.7)	550 (46.5)
Yes	10331 (34.3)	634 (53.5)
**Total METs per day, n (%)**		
Below average	22523 (74.8)	668 (56.4)
Above average (more active)	7607 (25.2)	516 (43.6)
**Alcohol consumption, n (%)**		
Never drinker	12484 (41.4)	391 (33.0)
Ever drinker	17646 (58.6)	793 (67.0)
**Comorbidities, n (%)**		
None	19372 (64.3)	733 (61.9)
One comorbidity	6376 (21.2)	171 (14.4)
Two or more comorbidities	4382 (14.5)	280 (23.6)
Obesity (BMI ≥ 30 kg/m^2)	983 (3.3)	43 (3.6)
Diabetes	1914 (6.4)	155 (13.1)
Hypertension	6521 (21.6)	281 (23.7)
Hyperlipidemia	4275 (14.2)	182 (15.4)
Cardiovascular disease	708 (2.3)	120 (10.1)
Cerebrovascular disease	387 (1.3)	104 (8.8)
Chronic kidney disease	490 (1.6)	124 (10.5)
Chronic hepatitis or cirrhosis	383 (1.3)	111 (9.4)
Chronic obstructive pulmonary disease	311 (1.0)	106 (9.0)
Asthma	1039 (3.4)	145 (12.2)
Malignant tumor	663 (2.2)	125 (10.6)
Immunodeficiency§	650 (2.2)	131 (11.1)
**Engagement in infection prevention measures, n (%)¶**		
Below average	11204 (37.2)	660 (55.7)
Above average (more careful)	18926 (62.8)	524 (44.3)
**SARS-CoV-2 vaccination status, n (%)**		
No or one time of mRNA vaccination	3911 (13.0)	256 (21.6)
Two or three times of vaccination	26219 (87.0)	928 (78.4)
**Year of infection, n (%)**		
Over one year prior to the survey	–	527 (44.5)
Within the preceding year	–	677 (57.2)
**Hospitalization due to COVID-19, n (%)**		
No	–	736 (62.2)
Yes	–	448 (37.8)
**Oxygen administration due to COVID-19, n (%)**		
No	–	745 (62.9)
Yes	–	439 (37.1)

Supplementary Table S6. Characteristics of participants (Unweighted). *Percentages in all participants (left column) / participants infected with SARS-CoV-2 (right column) (same as below). †21 prefectures that had been declared the State of Emergency more than once. ‡Under 18 years old. §Include use of corticosteroids, biologics, and immunosuppressive drugs. ¶Percentage of 12 infection prevention behaviors being performed. n: number, SARS-CoV-2: severe acute respiratory syndrome coronavirus 2, HTPs: heated tobacco products, METs: metabolic equivalents, BMI: body mass index, mRNA: messenger ribonucleic acid, COVID-19: coronavirus disease 2019.

	SARS-CoV-2 infection (n=1,184)			
	**n (%)[*]**	**Adjusted OR[†]**	**95%CI**	***p***
**Age**				
≤ 34 years	677 (7.85)	1 (reference)	–	–
35–49 years	317 (4.21)	**0.47**	0.40–0.55	<0.001
50–64 years	127 (1.86)	**0.22**	0.18–0.27	<0.001
≥ 65 years	63 (0.88)	**0.13**	0.09–0.18	<0.001
**Sex**				
Male	729 (4.95)	1.13	0.99–1.30	0.073
Female	455 (2.95)	1 (reference)	–	–
**Tobacco use status**				
Never-user	471 (2.75)	1 (reference)	–	–
Former user (combustible cigarettes or HTPs)	180 (2.77)	**1.25**	1.04–1.51	0.018
Current user of combustible cigarettes	86 (2.65)	1.09	0.86–1.40	0.478
Current user of HTPs	91 (6.83)	**1.97**	1.54–2.52	<0.001
Current dual user (combustible cigarettes and HTPs)	356 (18.18)	**4.07**	3.42–4.85	<0.001
**Occupation**				
Student / unemployed / retiree	174 (1.77)	1 (reference)	–	–
Healthcare worker	112 (4.58)	**1.33**	1.02–1.74	0.038
Remote worker	99 (4.46)	1.25	0.95–1.65	0.117
Other worker	799 (5.11)	**1.30**	1.07–1.57	0.009
**Household annual income**				
Below average	556 (3.84)	1 (reference)	–	–
Above average (higher)	452 (4.82)	1.09	0.94–1.26	0.265
Did not know / did not want to answer	176 (2.80)	0.90	0.75–1.08	0.261
**Education**				
Junior high school / high school	288 (3.49)	1 (reference)	–	–
University / technical school or more	896 (4.09)	0.94	0.81–1.09	0.426
**Housing tenure**				
Own house	640 (3.14)	1 (reference)	–	–
Rental house	502 (5.47)	1.09	0.94–1.26	0.251
Other (freeloader / rootless)	42 (7.02)	1.22	0.84–1.78	0.299
**Living in prefectures under alert[‡]**				
No	142 (2.19)	1 (reference)	–	–
Yes	1042 (4.40)	**2.02**	1.68–2.43	<0.001
**Household members other than the participant**				
None (living alone)	329 (4.86)	1 (reference)	–	–
Adult(s) only	427 (2.67)	0.91	0.77–1.09	0.302
Living with a child (children)[§]	428 (5.83)	**1.39**	1.16–1.67	<0.001
**Interacting with people living separately at least once a week**				
No	550 (2.78)	1 (reference)	–	–
Yes	634 (6.14)	**1.67**	1.47–1.90	<0.001
**Total METs per day**				
Below average	668 (2.97)	1 (reference)	–	–
Above average (more active)	516 (6.78)	**1.57**	1.38–1.78	<0.001
**Alcohol consumption**				
Never drinker	391 (3.13)	1 (reference)	–	–
Ever drinker	793 (4.49)	**1.17**	1.02–1.34	0.021
**Comorbidities[¶]**				
None	733 (3.78)	1 (reference)	–	–
One comorbidity	171 (2.68)	**1.31**	1.09–1.57	0.004
Two or more comorbidities	280 (6.39)	**2.73**	2.28–3.26	<0.001
**Engagement in infection prevention measures[‖]**				
Below average	660 (5.89)	1 (reference)	–	–
Above average (more careful)	524 (2.77)	**0.67**	0.59–0.77	<0.001
**SARS-CoV-2 vaccination status**				
No or one time of mRNA vaccination	256 (6.55)	1 (reference)	–	–
Two or three times of vaccination	928 (3.54)	**0.75**	0.64–0.88	<0.001

Supplementary Table S7. Factors associated with SARS-CoV-2 infection among all participants (n=30,130) (Unweighted). [*]Percentages of infected individuals in each category. [†]Estimated using multivariable logistic regression modeling with adjustment for all listed variables. Values of $p < 0.05$ were considered statistically significant and corresponding adjusted ORs are shown in bold. [‡]21 prefectures that had been declared the State of Emergency more than once. [§]Under 18 years old. [¶]12 diseases listed in Table 1. [‖]Percentage of 12 infection prevention behaviors being performed. SARS-CoV-2: severe acute respiratory syndrome coronavirus 2, n: number, OR: odds ratio, 95%CI: 95% confidence interval, HTPs: heated tobacco products, METs: metabolic equivalents, mRNA: messenger ribonucleic acid.

	Hospitalization due to COVID-19 (n=433)				Oxygen administration due to COVID-19 (n=424)			
	**n (%)***	**Adjusted OR†**	**95%CI**	***p***	**n (%)***	**Adjusted OR†**	**95%CI**	***p***
**Age**								
≤ 34 years	262 (39.6)	1 (reference)	–	–	264 (39.9)	1 (reference)	–	–
35–49 years	106 (33.9)	0.79	0.56–1.12	0.186	104 (33.2)	0.75	0.52–1.07	0.116
50–64 years	45 (35.4)	1.43	0.88–2.33	0.151	39 (30.7)	1.26	0.74–2.14	0.387
≥ 65 years	20 (32.3)	1.21	0.61–2.38	0.581	17 (27.4)	1.07	0.51–2.27	0.856
**Sex**								
Male	315 (44.4)	1.29	0.94–1.76	0.118	318 (44.8)	1.34	0.95–1.88	0.097
Female	118 (26.0)	1 (reference)	–	–	106 (23.3)	1 (reference)	–	–
**Tobacco use status‡**								
Never-user	105 (22.4)	1 (reference)	–	–	82 (17.5)	1 (reference)	–	–
Former user (combustible cigarettes or HTPs)	42 (23.9)	0.90	0.57–1.44	0.665	38 (21.6)	1.19	0.72–1.97	0.487
Current user of combustible cigarettes	28 (32.2)	1.11	0.63–1.97	0.717	29 (33.3)	1.76	0.98–3.19	0.061
Current user of HTPs	38 (44.7)	**1.78**	1.03–3.07	0.038	34 (40.0)	**2.00**	1.12–3.55	0.019
Current dual user (combustible cigarettes and HTPs)	220 (63.2)	**2.29**	1.56–3.36	<0.001	241 (69.3)	**4.50**	2.99–6.79	<0.001
**Comorbidities§**								
None	205 (28.4)	1 (reference)	–	–	198 (27.4)	1 (reference)	–	–
One comorbidity	58 (34.3)	1.12	0.73–1.71	0.598	50 (29.6)	0.82	0.52–1.31	0.410
Two or more comorbidities	170 (62.5)	**2.06**	1.42–2.99	<0.001	176 (64.7)	**1.95**	1.31–2.88	<0.001
**SARS-CoV-2 vaccination status‡**								
No or one time of mRNA vaccination	347 (56.1)	1 (reference)	–	–	352 (57.0)	1 (reference)	–	–
Two or three times of vaccination	86 (15.8)	0.66	0.40–1.11	0.120	72 (13.2)	0.89	0.49–1.61	0.704
**Year of infection**								
Over one year prior to the survey	321 (63.3)	1 (reference)	–	–	333 (65.7)	1 (reference)	–	–
Within the preceding year	112 (17.0)	**0.23**	0.14–0.38	<0.001	91 (13.9)	**0.14**	0.08–0.24	<0.001

Supplementary Table S8. Factors associated with hospitalization and oxygen administration due to COVID-19 among participants infected with SARS-CoV-2 once (n=1,164) (Unweighted). *Percentages of individuals who hospitalized (left column) / administered oxygen (right column) among those infected once in each category. †Estimated using multivariable logistic regression modeling with adjustment for all listed variables. Values of $p < 0.05$ were considered statistically significant and corresponding adjusted ORs are shown in bold. ‡Reflected the status at the time of the survey for infection within the preceding year and the status at one year prior to the survey for infection over one year prior to the survey. §12 diseases listed in Table 1. COVID-19: coronavirus disease 2019, n: number, OR: odds ratio, 95%CI: 95% confidence interval, HTPs: heated tobacco products, SARS-CoV-2: severe acute respiratory syndrome coronavirus 2, mRNA: messenger ribonucleic acid.

	All participants (n=30,130)		Participants infected with SARS-CoV-2 (n=1,117)	
	**Male (n=14,718)**	**Female (n=15,412)**	**Male (n=714)**	**Female (n=403)**
**Tobacco use status, n (%)***				
Never-user	5130 (34.9)	11080 (71.9)	136 (19.0)	244 (60.5)
Former user (combustible cigarettes or HTPs)	4324 (29.4)	2261 (14.7)	106 (14.8)	39 (9.7)
Current user of combustible cigarettes	2490 (16.9)	1081 (7.0)	66 (9.2)	25 (6.2)
Current user of HTPs	1054 (7.2)	504 (3.3)	45 (6.3)	30 (7.4)
Current dual user (combustible cigarettes and HTPs)	1721 (11.7)	485 (3.1)	360 (50.4)	65 (16.1)

Supplementary Table S9. Tobacco use status of male and female. *Percentages in all participants (left two columns) / participants infected with SARS-CoV-2 (right two columns) in each sex. n: number (weighted by inverse probability weighting), SARS-CoV-2: severe acute respiratory syndrome coronavirus 2, HTPs: heated tobacco products.

	Hospitalization due to COVID-19 (n=483)				Oxygen administration due to COVID-19 (n=461)			
	**n (%)***	**OR**	**95%CI**	***p***	**n (%)***	**OR**	**95%CI**	***p***
**Age**								
≤ 34 years	292 (45.6)	1 (reference)	–	–	290 (45.2)	1 (reference)	–	–
35–49 years	128 (44.9)	0.98	0.74–1.29	0.865	117 (41.1)	0.84	0.63–1.11	0.216
50–64 years	41 (38.0)	0.74	0.49–1.13	0.160	34 (31.5)	**0.55**	0.36–0.85	0.007
≥ 65 years	21 (33.9)	0.63	0.37–1.09	0.099	20 (32.3)	0.60	0.34–1.03	0.065
**Sex**								
Male	361 (52.0)	**2.48**	1.91–3.21	<0.001	357 (51.4)	**3.08**	2.35–4.03	<0.001
Female	123 (30.5)	1 (reference)	–	–	103 (25.6)	1 (reference)	–	–
**Tobacco use status**†								
Never-user	88 (23.3)	1 (reference)	–	–	63 (16.7)	1 (reference)	–	–
Former user (combustible cigarettes or HTPs)	45 (31.7)	1.51	0.98–2.31	0.060	42 (29.6)	**2.04**	1.30–3.20	0.002
Current user of combustible cigarettes	35 (37.6)	**1.99**	1.23–3.23	0.005	43 (46.2)	**4.28**	2.62–6.98	<0.001
Current user of HTPs	26 (37.7)	**2.03**	1.18–3.49	0.010	25 (36.2)	**2.92**	1.67–5.11	<0.001
Current dual user (combustible cigarettes and HTPs)	289 (69.5)	**7.48**	5.45–10.27	<0.001	288 (69.2)	**11.07**	7.87–15.57	<0.001
**Comorbidities**								
None	233 (35.0)	1 (reference)	–	–	217 (32.6)	1 (reference)	–	–
One comorbidity	63 (42.3)	1.36	0.95–1.95	0.097	47 (31.5)	0.94	0.64–1.38	0.769
Two or more comorbidities	188 (66.4)	**3.67**	2.73–4.92	<0.001	197 (69.6)	**4.71**	3.49–6.37	<0.001
Obesity (BMI ≥ 30 kg/m^2)‡	21 (51.2)	1.33	0.71–2.47	0.371	23 (56.1)	1.70	0.91–3.17	0.097
Diabetes	87 (56.5)	**1.75**	1.24–2.47	0.001	96 (62.3)	**2.61**	1.84–3.70	<0.001
Hypertension	178 (62.0)	**2.69**	2.04–3.56	<0.001	173 (60.3)	**2.77**	2.10–3.66	<0.001
Hyperlipidemia	119 (62.3)	**2.47**	1.79–3.41	<0.001	120 (62.8)	**2.79**	2.02–3.85	<0.001
Cardiovascular disease	102 (76.1)	**4.87**	3.21–7.40	<0.001	106 (79.1)	**6.71**	4.32–10.42	<0.001
Cerebrovascular disease	110 (79.7)	**6.05**	3.92–9.32	<0.001	114 (82.6)	**8.43**	5.32–13.35	<0.001
Chronic kidney disease	89 (67.9)	**3.07**	2.08–4.53	<0.001	96 (73.3)	**4.47**	2.97–6.71	<0.001
Chronic hepatitis or cirrhosis	104 (73.2)	**4.08**	2.76–6.04	<0.001	106 (74.6)	**5.01**	3.36–7.48	<0.001
Chronic obstructive pulmonary disease	102 (70.8)	**3.70**	2.52–5.43	<0.001	113 (78.5)	**6.47**	4.25–9.86	<0.001
Asthma	117 (67.2)	**3.07**	2.18–4.32	<0.001	122 (70.1)	**4.08**	2.87–5.79	<0.001
Malignant tumor	103 (69.1)	**3.27**	2.26–4.74	<0.001	124 (83.2)	**8.74**	5.59–13.64	<0.001
Immunodeficiency§	102 (76.7)	**5.03**	3.29–7.67	<0.001	91 (68.4)	**3.57**	2.41–5.27	<0.001
**SARS-CoV-2 vaccination status**†								
No or one time of mRNA vaccination	410 (61.7)	1 (reference)	–	–	405 (61.0)	1 (reference)	–	–
Two or three times of vaccination	73 (16.9)	**0.13**	0.09–0.17	<0.001	55 (12.7)	**0.09**	0.07–0.13	<0.001
**Year of infection**								
Over one year prior to the survey	384 (67.3)	1 (reference)	–	–	382 (66.9)	1 (reference)	–	–
Within the preceding year	99 (18.9)	**0.11**	0.09–0.15	<0.001	79 (15.0)	**0.09**	0.07–0.12	<0.001

Supplementary Table S10. Factors associated with hospitalization and oxygen administration due to COVID-19 among participants infected with SARS-CoV-2 once (n=1,097) (Univariable analysis). The ORs were estimated using univariable logistic regression modeling. Values of $p < 0.05$ were considered statistically significant and corresponding ORs are shown in bold. *Percentages of individuals who hospitalized (left column) / administered oxygen (right column) among those infected once in each category. †Reflected the status at the time of the survey for infection within the preceding year and the status at one year prior to the survey for infection over one year prior to the survey. ‡References are not having each comorbidity (same as the diseases below). §Include use of corticosteroids, biologics, and immunosuppressive drugs. COVID-19: coronavirus disease 2019, n: number (weighted by inverse probability weighting), OR: odds ratio, 95%CI: 95% confidence interval, HTPs: heated tobacco products, BMI: body mass index, SARS-CoV-2: severe acute respiratory syndrome coronavirus 2, mRNA: messenger ribonucleic acid.

References of Supplementary Information

1. Cabinet Secretariat. The State of Emergency. Available online at: https://corona.go.jp/emergency/ [accessed 22 June 2022] (2021).
2. Ainsworth, B. E., *et al.* 2011 Compendium of physical activities: a second update of codes and MET values. *Med Sci Sports Exerc.* **43**, 1575-1581. https://dx.doi.org/10.1249/MSS.0b013e31821ece12 (2011).
3. Gotanda, H., Miyawaki, A., Tabuchi, T. & Tsugawa, Y. Association between trust in government and practice of preventive measures during the COVID-19 pandemic in Japan. *J Gen Intern Med.* **36**, 3471-3477. https://dx.doi.org/10.1007/s11606-021-06959-3 (2021).
